# Genetic Diagnosis Using Whole Exome Analysis in Two Cases with Malignant Osteopetrosis of Infancy

**DOI:** 10.4274/jcrpe.2597

**Published:** 2015-12-03

**Authors:** Korcan Demir, Özlem Nalbantoğlu, Kadri Karaer, Hüseyin Anıl Korkmaz, Melek Yıldız, Selma Tunç, Behzat Özkan

**Affiliations:** 1 Dr. Behçet Uz Children’s Hospital, Clinic of Pediatric Endocrinology, İzmir, Turkey; 2 Dokuz Eylül University Faculty of Medicine, Division of Pediatric Endocrinology, İzmir, Turkey; 3 Intergen Genetics Center, Ankara, Turkey

**Keywords:** mutation, TCIRG1, CLCN7, osteopetrosis, whole exome analysis

## TO THE EDITOR

Malignant osteopetrosis of infancy (MOI) is a life-threatening form of osteopetrosis characterized by dense, sclerotic and fragile bones, impairment of bone marrow function, entrapment of cranial nerves, and growth retardation (1,2). The underlying mechanism leading to MOI is possibly an aberration in either the differentiation or function of osteoclasts. Until now, homozygous or compound heterozygous mutations in seven genes (TNFRSF11A, TNFSF11, TCIRG1, CLCN7, OSTM1, SNX10, and PLEKHM1) have been found in 80% of children with MOI (2). Detection of the exact cause and provision of genetic counselling via individual mutation analysis of all these genes would be expensive and time-consuming. Whole exome sequencing is being increasingly used given that its cost and the time needed for analysis are similar to that of single-gene sequencing (3,4). In addition, whole exome sequencing offers the probability to detect novel causative genes in the remaining 20% of patients with MOI.

Our first patient, a 9-day-old male infant, was referred to our institution with hypocalcemia (calcium, 5.4 mg/dL). His hemoglobin level was 8.6 g/dL and platelet count was 130 000/mm3. His parents were not relatives. There was no similar case in the family. His weight (3060 g, 25th-50th percentile), length (46 cm, 10th-25th percentile), and head circumference (36 cm, 10th percentile) measurements were normal, and no pathological examination finding was noted. Skeletal survey demonstrated sclerotic bones.

The second patient was a 7-day-old female infant with hepatosplenomegaly and hypocalcemia (calcium, 6.8 mg/dL). Her parents were relatives, and one of her elder brothers had died at age four months from complications of osteopetrosis without a genetic diagnosis. Physical examination revealed normal weight (3045 g, 25th-50th percentile), length (47.5 cm, 10th-25th percentile), and head circumference (37 cm, 25th percentile), and hepatosplenomegaly. She had thrombocytopenia (139.000/mm3) but no anemia or leukopenia. Radiographic findings revealed a dense skeleton, and the diagnosis of osteopetrosis was suggested.

**Figure 1:**
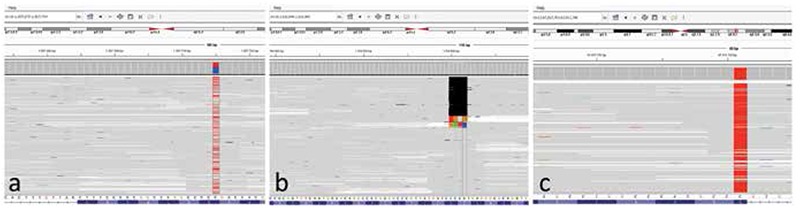
Partial sequences of the relevant regions of the patients (a) heterozygous c.718G>A mutation in exon 8 of CLCN7 gene, (b) heterozygous TTGG deletion (c.398_401delTTGG) in exon 5 of CLCN7 gene, (c) homozygous mutation c.2236C>T mutation in exon 18 of TCIRG1 gene

In these two patients, we employed whole exome analysis with particular attention to the seven candidate genes. The DNA samples of the patients were extracted from peripheral blood. Exome sequencing data generated in Genotypic (India) using HiSeq 2,500 sequencer were analyzed in the Intergen Genetics Centre. In the first case, 31.382 variants were detected. Among the possible causative genes, a novel heterozygous mutation (c.718G>A, p.Gly240Arg), which was strongly predicted to be a disease-causing mutation with in silico analyses with MutationTaster (mutationtaster.org), SIFT (sift.jcvi.org), PolyPhen-2 (genetics.bwh.harvard.edu/pph2), was detected in CLCN7 gene (Figure 1a). Whole gene MiSeq next-generation sequencing of CLCN7 confirmed the above-mentioned mutation and detected another novel frameshift mutation as well (Figure 1b), resulting in a compound heterozygous state (c.398_401delTTGG, p.Ile133Argfs*49 and c.718G>A, p.Gly240Arg). In the second case, 32.529 variants were detected. A previously reported homozygous nonsense mutation p.Gln746* (c.2236C>T) in TCIRG1 was detected and confirmed using MiSeq next-generation sequencing (Figure 1c). Genetic counselling was provided, and pre-implantation genetic testing was recommended for both families.

Whole exome analysis is a useful method for diseases in which multiple genes play a role in the etiology. However, it should be kept in mind that Sanger sequencing/next-generation sequencing may be needed when a heterozygous mutation is detected by whole exome sequencing in autosomal recessively inherited candidate genes.
